# Measuring Replicative Life Span in the Budding Yeast

**DOI:** 10.3791/1209

**Published:** 2009-06-25

**Authors:** Kristan K. Steffen, Brian K. Kennedy, Matt Kaeberlein

**Affiliations:** Department of Biochemistry, University of Washington; Department of Pathology, University of Washington

## Abstract

Aging is a degenerative process characterized by a progressive deterioration of cellular components and organelles resulting in mortality. The budding yeast *Saccharomyces cerevisiae* has been used extensively to study the biology of aging, and several determinants of yeast longevity have been shown to be conserved in multicellular eukaryotes, including worms, flies, and mice ^1^.  Due to the lack of easily quantified age-associated phenotypes, aging in yeast has been assayed almost exclusively by measuring the life span of cells in different contexts, with two different life span paradigms in common usage ^2^.  Chronological life span refers to the length of time that a mother cell can survive in a non-dividing, quiescence-like state, and is proposed to serve as a model for aging of post-mitotic cells in multicellular eukaryotes.  Replicative life span, in contrast, refers the number of daughter cells produced by a mother cell prior to senescence, and is thought to provide a model of aging in mitotically active cells. Here we present a generalized protocol for measuring the replicative life span of budding yeast mother cells. The goal of the replicative life span assay is to determine how many times each mother cell buds.  The mother and daughter cells can be easily differentiated by an experienced researcher using a standard light microscope (total magnification 160X), such as the Zeiss Axioscope 40 or another comparable model.  Physical separation of daughter cells from mother cells is achieved using a manual micromanipulator equipped with a fiber-optic needle.  Typical laboratory yeast strains produce 20-30 daughter cells per mother and one life span experiment requires 2-3 weeks.

**Figure:**
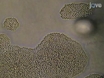


## Protocol

### Part 1: Prepare strains and plates for replicative life span analysis

This section describes the preparation of the solid YEPD plates for use in the replicative life span experiment and the preparation of yeast cells for life span analysis.

Using appropriate sterile technique, prepare YEPD agar plates (1% yeast extract, 2% bacto-peptone, 2% agar, 2% glucose) that will be used for culturing yeast cells and for replicative life span analysis. You should prepare at least 2 plates for every 4-5 strains to be analyzed in the life span experiment. Plates should be prepared at least 2 days before the life span experiment and allowed to dry before beginning the life span assay. If the experiment will not be initiated within 4-5 days of pouring the plates, they should be placed in a refrigerated incubator and wrapped in parafilm or plastic to prevent dessication.
Remove yeast strains from frozen stocks and streak for single colonies onto YEPD agar plates. Up to 6 strains can easily be streaked on the same YEPD plate by partitioning the plate into equally sized pie-shaped wedges.
Incubate the cells at 30°C for 2 days.
Remove cells from the incubator and patch cells from a single colony onto a fresh YEPD plate. Two patches from two different colonies should be generated for each strain. At this time, the strains to be analyzed should be coded to ensure the replicative life span experiment is performed "blind", where the dissectors do not know the identity of individual strains.
Incubate the patched, coded cells at 30°C until the following evening.
Remove the cells and lightly patch cells from each coded strain to fresh YEPD plates, which will serve as the experimental plates used for the replicative life span analysis. Cells should be patched along a vertical line on the left side of the YEPD plate (Figure 1). Approximately 3-5 patches per plate is optimal, assuming replicative life span analysis on 20 cells from each patch. It is not necessary (nor desirable) to transfer a large number of cells to the fresh YEPD plate, as this will cause the cells to grow thickly during the overnight incubation, which may limit their subsequent replicative life span.
Incubate the freshly patched plates overnight on the bench top.

### Part 2: Position cells for replicative life span analysis.

In this section we describe how to position the yeast cells on the plate and how to obtain virgin daughter cells for replicative life span analysis. From this point on, all plates should be parafilmed, except when undergoing dissection, in order to prevent dessication.

Using a microscope equipped with a microdissection apparatus suitable for yeast, transfer approximately 50 cells from the first patched strain to a position on the plate distal to the patched cells . If your microscope stage is graded, these gradations can be used to ensure the cells will be easy to find during subsequent iterations. On the Zeiss Axioscope 40, cells from the first patched strain can be placed at coordinates 90 x 10 and arrayed vertically from there.
Clean the needle of cells by repeatedly touching the agar surface, then create a hole in the agar by forcing the needle through the agar surface. This hole will serve as a marker to orient you on the plate during subsequent iterations of dissection and daughter cell removal. Be careful not to get yeast cells in the hole, or they will grow and form a colony.
Directly above the hole, vertically align individual yeast cells into 20 evenly spaced positions, with 1-3 needle diameters (~100 μM) between each cell position (Figure 1). Every few cells, place two cells adjacent to each other in the same position in the line rather than one. This allows for redundancy during virgin daughter cell selection, if one of the transferred cells fails to divide.
Between the 10^th^ and 11^th^ positions in the line, create a horizontal series of 7-8 holes in the agar. This will serve as a marker to orient you on the plate during subsequent iterations of dissection and daughter cell removal. Be careful not to get yeast cells in the holes, or they will grow and form a colony.
Repeat steps 2.1-2.4 for each patch on the plate, continuing to array cells vertically along the same axis. For step 2.2, the number of holes created should correspond to the patch number (one hole for the first patch, two holes for the second, etc.).
Once cells have been arrayed for each patch, parafilm the plate and incubate at 30°C for approximately 2 hours.
Repeat steps 2.1-2.6 for each plate in the experiment.

### Part 3: Obtain virgin daughter cells for life span analysis

In this section we initiate the life span analysis by ensuring that each cell starts as a virgin daughter cell.

Remove the plates from the incubator and verify that most of the arrayed cells have undergone a cell division. If additional time is needed to allow cell division to be completed, return the plate to the incubator.
Beginning with the first arrayed cell, use the needle to detach daughter cells from the mother cell by gently placing the needle on top of the attached cells. It is sometimes helpful to tap the side of the microscope base while the needle is resting on the cells.
Place the detached daughter cell in the vertical line, replacing the mother cell and any additional daughter cells and moving them temporarily aside.
Repeat step 3.2 and 3.3 for each of the arrayed cells. If a cell fails to divide, take a daughter cell from one of the redundant cells positioned in step 2.3. When finished, you should have 20 daughter cells for each patch aligned vertically on the life span plate.
Collect all of the mother cells and extra daughter cells into a pile and transfer them back to an area near the patches ("the graveyard"). It is important to keep unwanted cells far from the cells undergoing life span analysis to prevent formation of microcolonies and depletion of local nutrients.
Parafilm the plate and incubate at 30°C for approximately 2 hours.
Repeat steps 3.2-3.6 for each plate in the experiment.

### Part 4: Measuring the replicative capacity of individual cells

Prepare the life span data sheets. A life span data sheet can be a simple grid where each row corresponds to an individual mother cell and each column corresponds to an age-point (Figure 2). Label each data sheet according to the number of strains to be analyzed.
Remove the plates from the incubator and verify that most of the arrayed cells have undergone at least one cell division. If additional time is needed to allow cell division to be completed, return the plate to the incubator.
Beginning with the first arrayed cell on the first plate, visually observe the mother and daughter cell(s) and determine the number of cell divisions that have occurred. It is generally easy to determine how many daughter cells a mother has produced, based on characteristic patterns of cell arrangements. Record the number of daughter cells produced by the mother cell in the appropriate box on the life span score sheet.
Use the needle to detach daughter cells from the mother cell as described in step 3.2.
Retain the mother cell in its position in the vertical line and move the daughter cell(s) to temporarily aside.
Repeat steps 4.3-4.5 for each of the arrayed cells.
Collect all of the discarded daughter cells and move them to the 'graveyard', located near the original patches.
Parafilm the plate and incubate at 30°C for 2-3 hours.
Repeat steps 4.3-4.8 for each plate in the experiment.
Repeat steps 4.2-4.9 until all mother cells have stopped dividing. As the mother cells age, the cell cycle progression becomes slower and it will be desirable to incubate the plate for longer periods of time between age-points. Plates can be placed at 4°C overnight.

### Part 5: Representative Results.

The raw data produced by a replicative life span experiment is a list numbers corresponding to daughter cells produced by each mother cell at each age-point (Figure 2). By summing each row of the score sheet, the replicative life span for each mother cell is obtained. Data for mother cells from the same experimental group can be pooled to generate a survival curve (Figure 3A) and to perform statistical calculations, such as mean life span, median life span, and standard deviation. A non-parametric test, such as the Wilcoxon Rank-Sum test, can be performed to determine whether significant differences in life span were detected between experimental groups. When the experiment works correctly, the survival curve should be roughly consistent with Gompertz-Makeham kinetics showing a sigmoidal shap with low early mortality followed a relatively rapid decline in survival and a flattening out of the curve a later ages. Most wild-type strains have replicative life span values in the 20-30 generation range, although variation outside of this range has been reported.


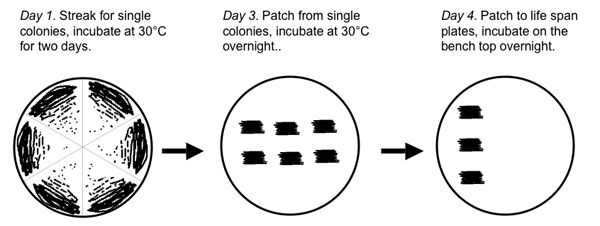
 
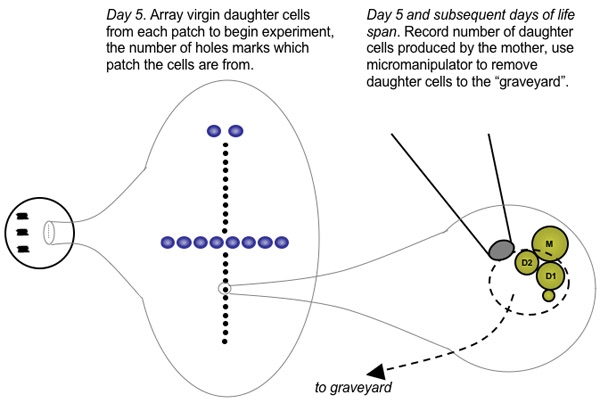
 **Figure 1. Diagram of a typical replicative life span plate**. The evening before beginning microdissection of yeast cells, 3-5 strains are lightly patched onto the surface of a YEPD plate (red boxes) in a vertical line along one side of the plate. Following growth overnight, approximately 30 individual cells from each strain are arrayed vertically into a line containing 20 positions. Life span analysis will be performed on v virgin daughter cells obtained from these initial cells. Extra cells obtained from microdissection during the experiment (e.g. unwanted daughter cells) are placed in "the graveyard" (gray oval), which is located far away from the experimental cells in order to avoid local depletion of nutrients as colonies are formed.


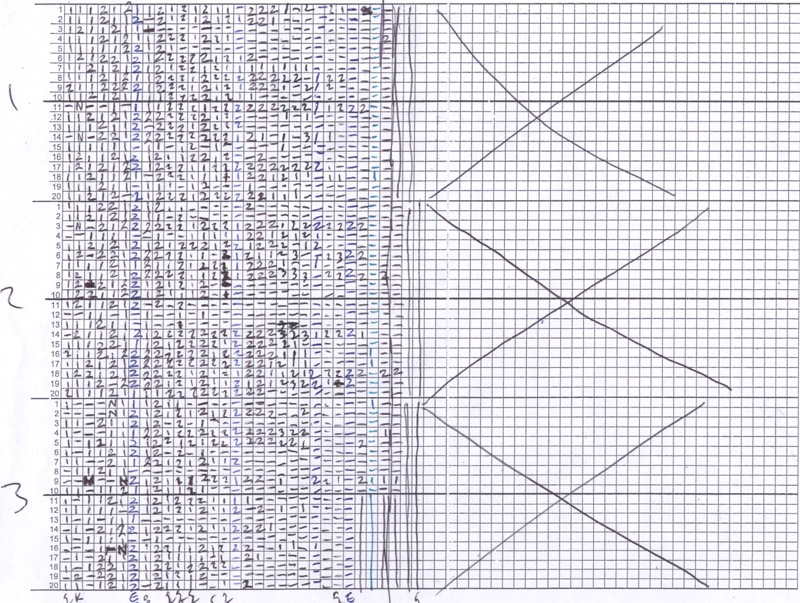
 **Figure 2. A replicative life span tally sheet.** Raw data from a replicative life span experiment is shown. Each row corresponds to a single yeast mother cell and each column corresponds to an age-point. The number entered in each box is the number of daughter cells produced by that mother cell at that age-point. Each strain is labeled with a coded number such that the individual dissecting the yeast cells has no knowledge of the identity of the strains being analyzed. Twenty cells are assayed for each strain.


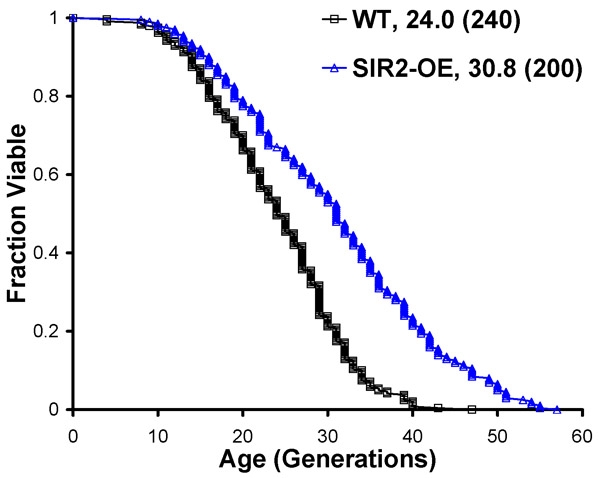
 **Figure 3. Representative survival and growth curves from a replicative life span experiment.** (A) Survival curves are obtained by plotting the fraction of mother cells still alive as a function of replicative age (number of cell divisions. (B) Growth curves are obtained by plotting the average number of daughter cells produced by a given strain as a function of age-point. In this example, strain #2 is longer-lived but is slower growing, as evidenced by the reduced initial slope of the growth plot.
